# Editorial: Post-Exercise Recovery: Fundamental and Interventional Physiology

**DOI:** 10.3389/fphys.2016.00003

**Published:** 2016-01-21

**Authors:** Sergej M. Ostojic

**Affiliations:** ^1^Biomedical Sciences Department, Faculty of Sport and Physical Education, University of Novi SadNovi Sad, Serbia; ^2^School of Medicine, University of BelgradeBelgrade, Serbia

**Keywords:** skeletal muscle, recovery, exercise, nutrition, autonomic response

Post-exercise Recovery: Fundamental and Interventional Physiology is a *Frontiers in Physiology* Research Topic aimed to provide new advances in the basic and applied study regarding recovery after exercise in the context of both physiological and pathophysiological conditions. During the past decade or so, a plethora of novel findings has been provided concerning attributes of post-exercise recovery in health and disease, and different physiological, medical, and nutritional interventions targeted to manage post-exercise recovery. Since 2000, more than 1200 peer-reviewed papers have been published in the leading biomedical journals describing the biology and medicine of post-exercise recovery. In this Research Topic, we presented perspectives, opinion pieces, and original research and review articles focused to understand a complex physiology of post-exercise recovery, and to outline recent experimental data from interventional studies.

Interpreting the physiology of time period immediately after exercise as an authentic biological phenomenon recently becomes of notable importance for both clinical exercise physiologists and sports scientists. Irregular or abnormal physiological responses during this time window might indicate much more than poor health or low fitness level (Terziotti et al., 2001; Lind et al., 2009; Hausswirth and Le Meur, 2011; Rattray et al.), with the physiology of recovery period should be investigated in a more distinctive and multidisciplinary manner. On this regard, Luttrell and Halliwill present an interesting perspective paper that conceptualizes post-exercise recovery as a discrete event from exercise itself. Authors discussed that post-exercise recovery might help us to identify and monitor several clinical conditions, being a perfect time window for therapeutic interventions in both athletic and clinical environment, and/or prognostic time span providing predictive information regarding sport performance and health outcomes. This novel concept encourages multi-faceted approach to the research field of “physiology of recovery” and merits further investigation.

Another new and compelling idea explored in this Research Topic is the specificity concept for the recovery after exercise. Minett and Costello present an opinion article on the need for post-exercise recovery protocols to be suitably matched and specifically prescribed to the individual and environmental characteristics and needs. Here, authors discuss the controversy surrounding the effectiveness of post-exercise cooling for recovery, suggesting more specific approach should be emphasized for this and other post-exercise interventions in future studies. Nutrition seems to be another important player in post-exercise recovery game, requiring more specific modus operandi. Specific timing of food ingestion, food content, and quality are some distinct factors that might dictate recovery dynamics (Howe et al., 2014). Recent studies have focused on post-exercise protein intake to enhance metabolic adaptations and maximize training adaptations (Pasiakos et al., 2014; Beck et al., 2015). On this regard, Morton et al. present a comprehensive overview of the current knowledge of the field on nutritional manipulation to augment resistance-training induced skeletal muscle hypertrophy during the recovery period. Importantly, a variety of nutritional factors affecting balance between muscle anabolism and catabolism are highlighted, and more specific information about protein and carbohydrate co-ingestion during recovery should arise from future studies.

Important players in post-exercise recovery are the cytokines, small proteins involved in cell signaling and apoptosis (Nieman and Pedersen, 1999). Cytokines, such as tumor necrosis factor-α (TNF-α) or interleukin 6 (IL-6), are critical in the regulation of inflammation, muscular injury, and repair after exercise (Philippou et al., 2012). In this Research Topic, Townsend et al. present an original article that compared the effects of different interventional recovery protocols administered following intense resistance exercise on circulating TNF-α and TNF-α receptor expression. Authors show favorable cytokines response after specific recovery intervention, implying that the efficacy of a recovery protocol depends on both the modality of the intervention and the particular time point of its administration during recovery. However, more research is needed concerning the identification of time frames in which the recovery interventions are most beneficial to muscle function and repair.

Can recovery rates from single- and multiple-bout exercise help us to better understand “physiology of recovery”? In their opinion article, Girard et al. advance a link between neural drive, environmental stress and recovery dynamics in repeated exercise, suggesting possible role of perceptual recovery to augment athletic performance. Same group present an original paper addressing the recovery of performance during repeated sprints in manipulated hypoxia (Girard et al.), providing insight about an association between external stressors and neuro-mechanical alterations during recovery. Disturbances in autonomic neural drive during post-exercise recovery are also prominent in clinical populations (Sheppard et al., 2007; Minai et al., 2012; Florea and Cohn, 2014). Here, Trevizani et al. present an original paper that describes cardiac autonomic recovery after single resistance exercise session in hypertensive men. The concept that exercise promotes neuromuscular modulation can be a link between exercise and its performance- and health-related effects. In addition, Akazawa et al. report an original research about the effects of regular aerobic training on post-exercise reduction of blood pressure in post-menopausal women, suggesting the clinical importance of monitoring central hemodynamic responses after exercise.

In addition to all these outstanding contributions, this research topic also presents captivating original articles evaluating how low-volume high-intensity training affects tissue-specific circulating microRNAs as related to conventional biochemical and performance indices (Cui et al.); and the correlation of non-invasive recovery measures, heart rate (HR) variability and post-exercise HR recovery, with increases in physical activity and cardiorespiratory fitness in non-exercising women (Tonello et al.). Both articles support the use of novel or improved markers of post-exercise recovery that may provide new insights into the physiological perturbations that occur in the body during and after exercise.

Besides presenting contemporary knowledge and viewpoints on the issue of post-exercise recovery, hopefully this Research Topic will further advance the field of “physiology of recovery” by generating innovative ideas, multidisciplinary approaches and experimental studies.

## Author contributions

Paper concept and design, drafting the paper, critical revision of the paper for intellectual content: SO.

## Funding statement

This study was supported by the Serbian Ministry of Education, Science and Technological Development (Grant No. 175037), and the Faculty of Sport and Physical Education, University of Novi Sad (2015 Annual Award).

### Conflict of interest statement

The author declares that the research was conducted in the absence of any commercial or financial relationships that could be construed as a potential conflict of interest.
